# Exploring multimodal collaborative storytelling with Pepper: a preliminary study with zero-shot LLMs

**DOI:** 10.3389/frobt.2025.1662819

**Published:** 2025-10-08

**Authors:** Unai Zabala, Juan Echevarria, Igor Rodriguez, Elena Lazkano

**Affiliations:** Department of Computer Science and Artificial Intelligence, University of the Basque Country (EHU), Donostia, Spain

**Keywords:** collaborative storytelling, social robotics, zero-shot LLM, gesture generation, human-robot interaction

## Abstract

With the rise of large language models (LLMs), collaborative storytelling in virtual agents or chatbots has gained popularity. Despite storytelling has long been employed in social robotics as a means to educate, entertain, and persuade audiences, the integration of LLMs into such platforms remains largely unexplored. This paper presents the initial steps for a novel multimodal collaborative storytelling system in which users co-create stories with the social robot Pepper through natural language interaction and by presenting physical objects. The robot employs a YOLO-based vision system to recognize these objects and seamlessly incorporate them into the narrative. Story generation and adaptation are handled autonomously using the Llama model in a zero-shot setting, aiming to assess the usability and maturity of such models in interactive storytelling. To enhance immersion, the robot performs the final story using expressive gestures, emotional cues, and speech modulation. User feedback, collected through questionnaires and semi-structured interviews, indicates a high level of acceptance.

## 1 Introduction

Storytelling has been an essential form of communication throughout human history, serving as a means of education, entertainment, and persuasion. It plays a fundamental role in shaping culture, transmitting knowledge, and fostering social connections. In recent years, the integration of storytelling into Human-Robot Interaction (HRI) has opened new possibilities for engaging and meaningful interactions, particularly in fields such as education (Zhang C. et al., 2024), therapy (Alonso-Campuzano et al., 2024), and entertainment (Nichols et al., 2021). Social robots, with their ability to recognize speech, process language, and express emotions, are becoming increasingly sophisticated tools for presenting stories to listeners. Their physical presence can enhance engagement, making storytelling experiences more immersive and impactful compared to purely virtual or digital systems (Belpaeme et al., 2018; Fiske et al., 2019; Rasouli et al., 2022).

The rise of Large Language Models (LLMs) has significantly influenced HRI by equipping robots with advanced Natural Language Processing (NLP) capabilities, enabling them to generate original and contextually appropriate narratives in real-time. Unlike traditional scripted approaches that rely on preprogrammed dialogue, LLM-based storytelling may allow robots to dynamically adapt to user input, making interactions more flexible, immersive, and responsive. Beyond generating narratives, LLMs also should facilitate collaborative storytelling, transforming the robot from a passive narrator into an active co-creator. By integrating user suggestions, expanding ideas, and maintaining narrative coherence, these models foster more immersive and interactive storytelling experiences, enhancing creativity, engagement, and personalization.

Previous research in collaborative storytelling with social robots has primarily relied on LLMs that are fine-tuned for the specific task of storytelling (Nichols et al., 2020; Harmon and Rutman, 2023). The primary advantage of storytelling systems that use LLMs over traditional approaches lies in the enhanced naturalness and flexibility with which users can interact with the robot. Such systems allow users to express themselves in an unconstrained manner, with the scope for interaction limited primarily by the user’s own imagination. However, current LLM-based storytelling systems exhibit several limitations. Notably, interactions remain predominantly turn-based, and inference times can be prohibitively long, which may hinder the fluidity of real-time engagement. These challenges highlight areas for further research and optimization in the development of more responsive and interactive storytelling agents, showing significant room for innovation in the context of social robotics.

The objective of this preliminary study is to present the initial steps toward a multimodal collaborative storytelling system in which users co-create stories with the social robot Pepper. This is achieved through natural language interaction and the presentation of physical objects, which the robot recognizes and incorporates into the narrative. The main contributions of this work can be summarized as follows:

• The design and implementation of a multimodal collaborative storytelling system architecture for the social robot Pepper, integrating natural language interaction, vision, speech, and expressive behavior.
• The incorporation of Llama 2 for zero-shot story generation and adaptation, together with YOLOv11-based object recognition, enabling users to influence the narrative both verbally and by presenting physical objects.
• The integration and orchestration of complementary modules, including text-to-speech (Nuance’s TTS), automatic speech recognition (Whisper), and emotion recognition (RoBERTuito), within an embodied social robot designed to enhance the expressiveness and immersion of human–robot storytelling interactions.
• An experimental evaluation combining standardized (UEQ), custom questionnaires, and qualitative analysis (Affinity Diagram) to capture user feedback on naturalness, engagement, and overall acceptance of the system.

## 2 Related work

Storytelling is a powerful communicative tool that supports language acquisition in children while fostering critical thinking, self-esteem, and cultural awareness in educational contexts (Davidhizar and Lonser, 2003; Wright, 1995). With technological advancements, storytelling has evolved from oral and written traditions to digital formats enriched with multimedia elements such as text, images, sound, and video (Alismail, 2015). Within this digital framework, tools like Storybird have been employed to enhance foreign language students’ writing skills by promoting creativity, vocabulary development, and grammatical accuracy through the design of engaging narrative projects (Castillo-Cuesta et al., 2021). While digital storytelling (DST) has traditionally relied on virtual platforms and video-based systems (Essien and Parbanath, 2024), recent developments in LLMs and social robotics opened new possibilities for more interactive and adaptive storytelling experiences.

LLMs have significantly influenced storytelling by enabling coherent and adaptive text generation, eliminating the rigid constraints of scripted narratives. In some recent studies, such as Harmon and Rutman (2023), authors have explored how different open-source LLMs handle narrative dilemmas by analyzing their ability to generate logical story continuations based on varied prompts. Their study highlights the impact of prompt engineering in shaping the consistency and creativity of AI-generated stories. Similarly, Venkatraman et al. (2024) introduced CollabStory, a dataset where multiple LLMs collaboratively generate narratives each contributing with different segments of a story, demonstrating the potential of multi-model storytelling. Expanding on multimodal storytelling approaches, (Yotam et al., 2024), developed a system where children’s drawings are used as prompts for a GPT-4-based model to generate character descriptions and background narratives, allowing for interactive and visually-driven storytelling. These studies showcase how LLMs enable more interactive and flexible storytelling, adapting to user input and diverse formats.

Building on these advances, recent research has begun to explore how LLMs can also drive storytelling in immersive environments such as virtual or augmented reality. On the one hand, systems like Aisop explicitly frame the LLM as the storyteller, autonomously generating and narrating stories in virtual reality while complementing the narrative with speech and visual renderings (Gatti et al., 2024). On the other hand, LLMs have also been embedded within interactive agents, as demonstrated in recent work that deployed a GPT-4-based character in VRChat, capable of producing contextually appropriate verbal responses as well as coordinated gestures and facial expressions (Wan et al., 2024). Together, these approaches highlight both the narrative potential of LLMs and their capacity to embody interactive roles in virtual environments, pointing to broad opportunities for human-AI interaction through storytelling.

Beyond purely digital environments, social robots have emerged as an alternative platform for storytelling, leveraging embodiment and multimodal engagement to enhance interaction, making storytelling experiences more immersive. For instance, Liang and Hwang (2023) conducted a comparative study on robot-based vs. video-based DST systems for English as a foreign language learners. Their results demonstrated that robot-assisted storytelling improved English-speaking skills, narrative engagement, and communication confidence, outperforming traditional video-based methods. Similarly, Chang et al. (2023) explored the use of robot-assisted storytelling in pediatric healthcare, demonstrating that robots were more effective than video-based health education methods in reducing children’s anxiety during medical procedures. Additionally, their findings indicate that robotic interventions enhanced emotional expression and strengthened therapeutic engagement, further highlighting their advantages in healthcare settings. Although not directly compared to video-based methods, the work of Alimardani et al. (2021) explored the use of the NAO robot in therapeutic interventions for children with Autism Spectrum Disorder (ASD). The robot tells short, one-sentence stories while expressing emotions through body language and LED color cues, helping children improve emotional recognition and social interaction skills. This highlights the potential of robotic storytelling as a tool for therapy and emotional development.

Building on both LLM-driven storytelling and robot-assisted storytelling, collaborative storytelling introduces an interactive approach where the story evolves dynamically through user-robot interaction. There are few studies that integrate LLMs into collaborative storytelling with social robots, leaving a largely unexplored area of research. A few notable examples identified in the literature include the work of Elgarf et al. (2022), who developed two storytelling models (one creative and one non-creative) to analyse their impact on children’s engagement. Their findings suggest that the creative model enhanced children’s imagination, though the mode of interaction (robot-led or child-led) did not significantly alter the outcomes. Nichols et al. (2020), Nichols et al. (2021) introduced a turn-based collaborative storytelling system with the Haru robotic head, where storytelling is initiated from a database and progresses through user input and LLM-generated continuations, allowing for a more interactive and engaging storytelling experience.

In contrast to previous research, our approach uses zero-shot LLMs, avoiding the need for fine-tuning or multiple specialized models, while still enabling coherent, real-time storytelling. While such models can yield improved task-specific responses, this approach often comes at the expense of generalization capabilities, thereby limiting the adaptability of the system and constraining future extensions of the activity. Combined with multimodal interaction through natural language and object-based input, this results in a novel and streamlined framework for dynamic story co-creation with social robots. Furthermore, embodied agents showed up as a powerful alternative to wearables as it is closer collaborative storytelling between two humans.

## 3 Collaborative storytelling: Behavior generation

The storytelling process begins with the user requesting a story, to which the robot responds by offering a selection of five classic fairy tales. After the user selects one, the robot reads the story aloud without enacting it, dividing it into the three act structure: Setup, Confrontation, and Resolution. At the end of each segment, the user is given the option to personalize the story, submitting requests for changes of any type, given the unrestricted nature of the modification system. Once all modifications have been gathered, the robot proceeds to perform the adapted version of the story, integrating both verbal and non-verbal expressiveness.

The core of the proposed approach can be summarized in three main steps: (1) story selection and reading, (2) story adaptation and integration of user input, and (3) final story enactment.

**FIGURE 1:**
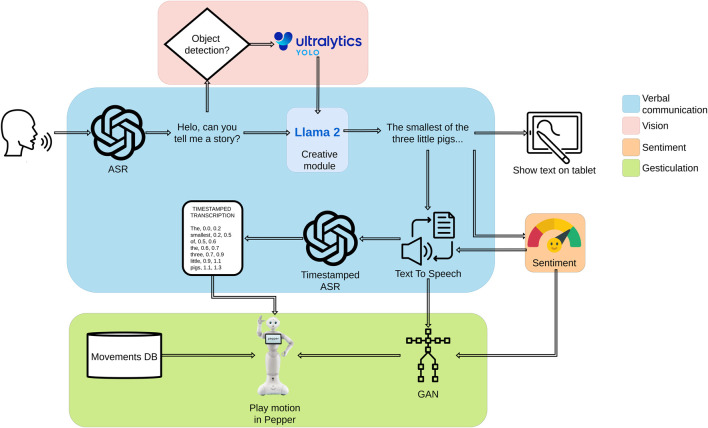
System overview.

The following subsections provide a detailed description of these steps, along with an overview of the different modules that compose the robot’s architecture, including the creative module as well as, the verbal communication, vision, sentiment analysis, and gesticulation modules. To better understand how these elements are interconnected, Figure 1 presents an overview of the system.

### 3.1 Choosing and introducing the story

The creative module is responsible for producing and structuring the story texts that serve as the foundation for the narrative.

When deploying large language models (LLMs) for specialized applications, two common strategies are contextual adaptation and one-shot or few-shot prompting. Contextual adaptation consists of enriching the model’s input with domain-specific knowledge, thereby facilitating more accurate and relevant responses. Conversely, one-shot and few-shot prompting provide exemplar demonstrations of the desired task within the input prompt, which improves the model’s ability to generalize the expected output structure and task-specific behavior. Since collaborative storytelling requires multiple instructions that are difficult to capture in a few examples, it was decided to use the context modification technique.

The LLM was instructed to emulate Pepper, an interactive storytelling robot, and respond to a story request by letting it presenting a list of five children’s stories (showing the title and the author). Although the LLM is responsible for generating the five stories, this process is inherently non-deterministic due to the stochastic nature of language model outputs. Therefore, the resulting list may vary across different executions, even with the same prompt (see Table 1). Once the user selected a story, the model narrated it dividing the story in Setup, Confrontation, and Resolution.

**TABLE 1:** List of stories selected by Llama2 and the times they have been proposed to the user. Others are other stories that appear just once.

Story name	Num. Appearances
Where the Wild Things Are by Maurice Sendak	23
The Very Hungry Caterpillar by Eric Carle	23
The Giving Tree by Shel Silverstein	21
Corduroy by Don Freeman	22
The Velveteen Rabbit by Margery Williams	10
Make Way for Ducklings by Robert McCloskey	4
Charlotte’s Web by E.B. White	4
Llama Llama Red Pajama by Anna Dewdney	3
The Lion, the Witch and the Wardrobe by C.S. Lewis	2
Others	13

An evaluation was conducted to identify the most suitable LLM for this task, considering three locally deployable models: Llama 2 13B, Vicuna 13B, and Mistral 7B v0.1, all utilizing 4-bit Activation-aware Weight Quantization (AWQ) (Lin et al., 2024). To compare their performance, an objective assessment was carried out, measuring video memory usage and inference speed, as detailed in Table 2. The evaluation was performed on two GPUs (RTX 2080Ti 11 GB and RTX 4080 16 GB), ensuring that all models met the minimum memory and processing requirements.

**TABLE 2:** Memory usage of different LLM models and mean inference time.

Name	Memory (MiB)	Speed (token/s)
Llama 2 13B Chat AWQ	9358	∼18
Mistral 7B v0.1 AWQ	7140	∼89
Vicuna 13B Chat AWQ	9642	∼20

Llama 2 13B^1^ was ultimately selected for two key reasons. First, its original model was optimized for dialogue-based tasks, making it a better fit for interactive storytelling compared to Mistral 7B v0.1, which is primarily optimized for reasoning and coding tasks. Second, when compared to Vicuna 13B, which also specializes in chat-based interactions and shows similar performance, Llama 2 13B showed higher creativity in generating modified story outputs.

To capture the user’s requests, the system employs Whisper^2^ as the Automatic Speech Recognition (ASR). In this step, Whisper processes the spoken input, transcribing the user’s request into text. This transcribed text is then passed to the Creative Module, which interprets the request and generates an appropriate response following the rules defined in the context.

Once the creative module produces a text-based response, it is processed by the TTS module, which converts it into natural-sounding speech. In this step, the robot does not need to enact but only “read” the response and thus, a neutral voice was selected and converted to audio using Nuance’s TTS tool integrated in Pepper for fast speech synthesis.

### 3.2 Personalizing the story

Personalization requires the system to process the modifications introduced by the user and to reconstruct the narrative to ensure coherence and logical flow. As mentioned before, these modifications are gathered while the robot completes the narration of the original version of the story, and only after this end enacts the final personalized story.

To achieve this, various key modules are involved. The creative module, powered by the Llama 2 model, integrates user modifications into the storyline, adapting characters, scene descriptions, etc. accordingly. Additionally, as the system is designed for multimodal interaction, it incorporates both speech and vision-based inputs. The ASR module captures user-proposed modifications through voice commands, ensuring seamless verbal interaction. Meanwhile, the Vision Module allows the user to introduce visual elements into the story. By showing objects to the robot, users can further personalize the narrative.

The robot relies on the Ultralytics YOLOv11^3^ model for object recognition. Since the model is used without fine-tuning, only objects belonging to categories that YOLOv11 can already classify are considered during the interaction. To confirm successful recognition, an image of the detected object is displayed on Pepper’s tablet.

### 3.3 Bringing the story to life

Finally, the robot performs the adapted version of the story using expressive speech, synchronized gestures, and emotional modulation, creating a more natural and immersive storytelling experience. This process integrates gesture generation, emotion display, and speech adaptation, ensuring that the robot conveys the narrative with greater naturalness and engagement.

The gesticulation module is responsible for generating body gestures that enhance the robot’s expressive storytelling. It follows a hybrid approach that combines beat-like gestures with semantically related gestures. Beat-like gestures, which provide rhythm and emphasize the prosody of speech, are synthesized using a Generative Adversarial Network (GAN) trained on motion capture data of people speaking Zabala et al. (2020). The GAN takes speech duration as input and produces sequences organized into Units of Movement (UM), each composed of four consecutive poses. However, beat gestures alone are insufficient for fully expressive storytelling. To address this, semantically related gestures, such as deictic, metaphoric, and iconic movements, are incorporated through a gesture database linked to specific lexemes. These gestures are sourced both from NAOqi’s library of predefined animations and from custom-designed motion sequences. When multiple relevant lexemes appear in a sentence, a probabilistic model governs selection to balance gesture distribution and avoid repetition by favoring less frequently used gestures. For precise temporal alignment, Whisper is used to transcribe the speech and determine the onset of each target word; gestures are scheduled to begin one motion unit before the detected start of the corresponding word, resulting in more natural and synchronized gesture execution (see (Zabala et al., 2022) for more details).

Given Pepper’s limited facial expressiveness, alternative modalities such as body language, speech modulation, and LED-based visual cues are employed to effectively convey emotions. To analyse sentiment in the LLM-generated sentences, a RoBERTuito-based model (Pérez et al., 2021) is used to produce a normalized sentiment vector with negative, neutral, and positive values. These values are mapped to a continuous scale between 0 (negative), 1 (neutral), and 2 (positive), as shown in Equation 1.

$$S_{\mathrm{new}}=S_{\mathrm{neu}}*1+S_{\mathrm{pos}}*2$$ (1)

The extracted sentiment influences multiple expressive channels. Body language is modulated by adjusting the speed of movement through the temporal spacing between poses in each Unit of Movement (UM). Simultaneously, speech rate and pitch are dynamically adapted to reflect the emotional tone of the story. Additionally, Pepper’s eye color is used as a visual indicator of sentiment, with the color selection inspired by Plutchik’s wheel of emotions (Plutchik, 1980): yellow represents positive emotions, blue indicates negative ones, and white is used for neutral sentiment.

## 4 Experimental setup

We conducted a user study to assess the robot’s ability to engage users, integrate their modifications into the narrative, and deliver an expressive and coherent storytelling performance.

The demographic characteristics of the 25 participants are summarized in Table 3. This table categorizes participants by age and gender, with designations for total (T), male (M), and female (F) populations. For each demographic subgroup, the level of experience within the Knowledge Domain (KD) is reported separately for both Robotics (R) and Large Language Models (LLMs) (L). The last row (Total) of the table provides a comprehensive overview of the entire participant cohort.

**TABLE 3:** Population details. The KD levels are denoted as Z (zero knowledge), I (interacted with), W (worked with), and D (developed).

Age	T	M	F	KD	Knowledge level			
					Z	I	W	D
20–29	15	10	5	R	6.6%	40%	26.6%	26.66%
				L	0%	33.3%	53.3%	13.33%
30–39	4	2	2	R	25%	75%	0%	0%
				L	25%	25%	50%	0%
40+	6	4	2	R	16.6%	66.6%	16.6%	0%
				L	16.6%	50%	33.3%	0%
Total	25	16	9	R	12%	52%	20%	16%
				L	8%	36%	48%	8%

**FIGURE 2:**
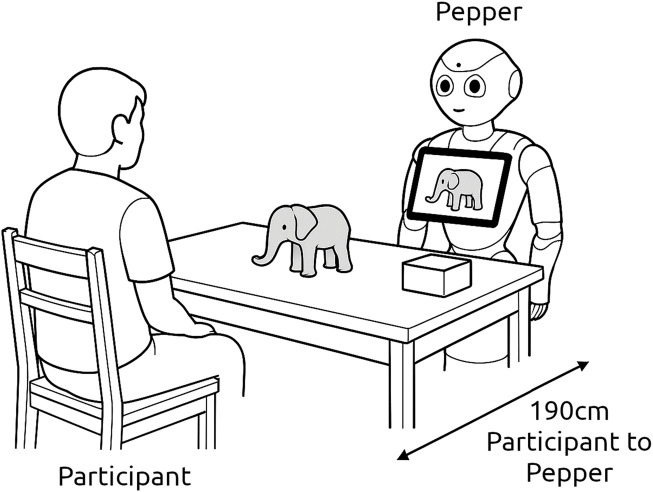
Diagram of the experimental setup.

**FIGURE 3:**
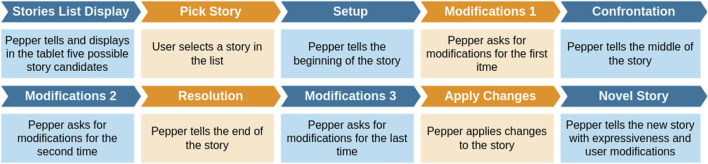
Interaction flow with Pepper.

Participants were seated facing the robot, with a table positioned between them. A box with multiple objects was placed on the table, providing participants with tangible tools to intuitively modify the story by incorporating physical elements into the narrative (see Figure 2). At the beginning of the session, the robot introduced itself and explained the experiment’s rules, ensuring participants fully understood the interaction process. Two different voices were used: a neutral voice generated with Google’s gTTS for the experiment narration, and a Nuance’s voice for telling the story. The storytelling system was activated upon the participant’s request, initiating the interaction as the robot guided them through story selection and modification, facilitating engagement and personalization. Figure 3 shows an overview of the interaction flow.

Right after the experiment, the user was asked to fulfil two questionnaires: the User Experience Questionnaire (UEQ) (Laugwitz et al., 2008), designed to measure user experience; and a Self-Designed Questionnaire (SDQ), designed to asses the quality of the robot’s behavioral development (see Table 4). The questionnaire gathers information across different key areas, such as user background, aspects related to the LLM, voice and body expression, gameplay experience, and overall satisfaction. After submitting both questionnaires, the users were asked for informed consent to record a brief semi-structured interview (audio only), covering topics such as object usage, interaction experience, and differences between the original and modified story presentations. The responses were later analysed using the Affinity Diagram method (Lucero, 2015), a technique for organizing abstract concepts into meaningful relationships to extract objective insights from unstructured data.

**TABLE 4:** Self-Designed Questionnaire content.

Question	Type
Aspects related to the LLM	
Q4: Were the proposed changes applied in the final version of the story?	Yes/No
Q5: The robot asks questions about possible changes to the story while telling the tale in order to make the changes at the end. Do you think this format is correct?	Yes/No
Q6: Does Pepper succeed in telling the story?	Yes/No
Q7: Do you think the system is creative?	Yes/No
Q8: Is the system consistent	Yes/No
Voice expression	
Q9: How many voices did you identified?	1, 2, 3, More
Q10: What emotions did you identify?	5 multi-choice
Body expression	
Q11: The body gesturing accompanies the emotions?	5 point Likert
Q12: The body gesturing accompanies the semantic of the text?	5 point Likert
Gameplay experience	
Q13: How well did Pepper keep you engaged throughout the story?	5 point Likert
Q14: Did you find the story entertaining and amusing?	5 point Likert
Q15: Did you find the storytelling system easy to understand and to interact with?	5 point Likert
Q16: Is the interaction flow adequate?	5 point Likert
Q17: Did you find the information displayed on the robot’s tablet useful?	5 point Likert
Global satisfaction	
Q18: Evaluate your global experience playing with Pepper	5 point Likert

## 5 Results

### 5.1 User experience questionnaire

**FIGURE 4:**
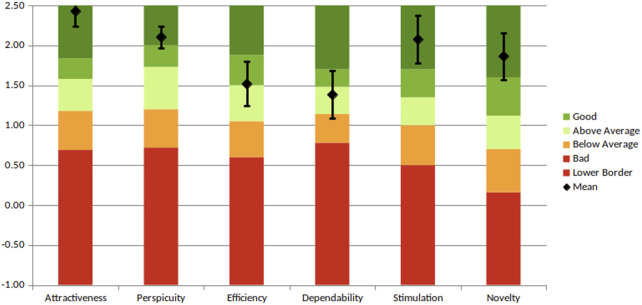
UEQ results for attractiveness, perspicuity, efficiency, dependability, stimulation and novelty.

Table 5 and Figure 4 present the system’s user experience results based on the UEQ benchmark. The outcomes are rated as excellent for attractiveness, perspicuity, stimulation, and novelty; good for efficiency; and above average for dependability. However, these promising results should be interpreted with caution, as the Cronbach’s Alpha coefficient does not provide strong support. Due to the small sample size, minor variations in user responses led to significant fluctuations in the reliability estimates.

**TABLE 5:** User Experience Questionnaire score, SD and Cronbach’s Alpha-Coefficient values.

Category	UEQ score	SD	Chronbachs alpha
Attractiveness	2.429	∓0.510	0.88
Perspicuity	2.106	∓0.355	−0.50
Efficiency	1.519	∓0.724	0.69
Dependability	1.385	∓0.785	0.55
Stimulation	2.077	∓0.767	0.74
Novelty	1.865	∓0.766	0.66

### 5.2 Self-designed questionnaire

Regarding the questionnaire defined to asses the quality of the developmental state of the robot’s behavior, analysis of LLM-related queries revealed that 88% of user-requested modifications were successfully implemented while maintaining narrative coherence. Experimental log files were preserved for subsequent analysis. These records suggest that 12% of participants reported unsuccessful application of their requested changes, primarily attributed to deficiencies in the speech recognition system. The ASR component exhibited imprecise transcription of user speech in 30.3% of instances. Notably, four subjects experienced suboptimal transcription in three out of five interactions. This ASR limitation also resulted in one participant reporting a complete failure in story narration by the Pepper robot. Regarding system performance evaluation, 96% of participants perceived the system as creative. Furthermore, all subjects (100%) affirmed the system’s consistency and endorsed the experimental design, which incorporated iterative interactions during the original narrative followed by the application of modifications in a novel version.

The analysis of voice expression revealed that the majority of participants accurately distinguished between the two distinct voices used in the experiment: the narrator and the storyteller. However, 28% of the participants identified three voices, likely due to confusion caused by tonal variations in the voice of the storyteller perceived as a separate entity. Only one participant identified a single voice. Regarding emotional recognition, happiness (22 responses) and sadness (10 responses) were the most frequently identified emotions, followed by anger and disgust (3 responses each) and fear (2 responses). Examination of the log files from the sentiment analysis module indicated that 217 sentences (53.06%) were classified as positive, 123 sentences (30.07%) as negative, and 69 sentences (16.87%) as neutral. This distribution suggests an imbalance in the perception of positive versus negative emotions.

Regarding body expression, users gave an average rating of 3.72 out of 5 to the body gesture/emotion correlation, and 4.24 out of 5 on the gesticulation/text semantic interdependence.

The overall gameplay experience was evaluated positively by participants. The robot, Pepper, effectively engaged users, achieving a mean score of 4.28 out of 5. The participants rated the experience as entertaining and enjoyable, with an average score of 4.44 out of 5. The system was perceived as intuitive and easy to interact with, receiving a score of 4.64 out of 5. Additionally, the interaction flow was deemed appropriate, with a mean score of 4.36 out of 5, and the information displayed on the robot’s tablet was considered highly useful, achieving the highest score of 4.84 out of 5. Overall, the global experience was rated very positively, with an average score of 4.56 out of 5.

The two-way random-effects, average-measures intraclass correlation coefficient (ICC) was computed (Koo and Li, 2016), specifically ICC(2,k), to assess the reliability of the average ratings across raters. The results indicated good to excellent reliability, ICC(2,k)=0.87,95%CI[0.70,0.97] . The associated F-test (F(7,168)=8.78,p=3.512665e−09<0.001) confirmed that the reliability is statistically greater than zero. Overall, these results demonstrate a high degree of agreement among raters.

### 5.3 Affinity diagram

**FIGURE 5:**
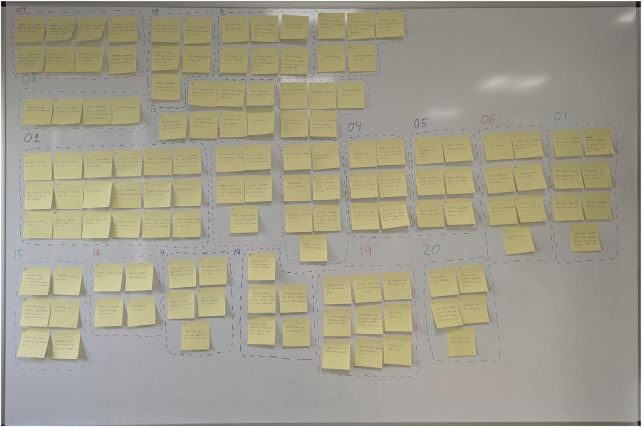
Sticky notes with annotations from the semi-structured interview.

**FIGURE 6:**
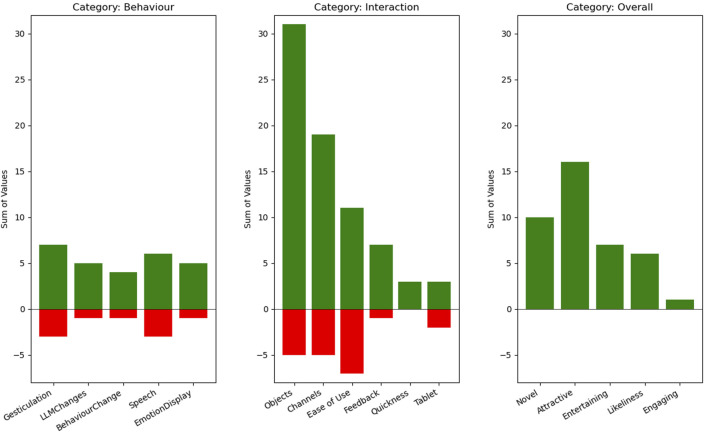
Results of the affinity diagram.

As mentioned earlier, a semi-structured interview was conducted immediately after the experiment. These interviews were later transcribed, and participants’ comments were annotated using sticky notes for analysis (see Figure 5). During the interviews, participants were asked to evaluate the use of physical objects as an interaction modality, describe their overall experience with the system, and provide a general assessment of the interaction. They were also invited to reflect on any perceived differences between the original and modified versions of the story beyond the narrative content. Finally, participants were encouraged to suggest possible improvements to enhance the system’s functionality and user experience. Subsequently, these notes were grouped into clusters following the criteria of three non-participant researchers, yielding three primary categories (behavior, interaction, and overall experience) and a total of 16 subcategories (see Figure 6). This process facilitated systematic organization and analysis of the qualitative data collected from the interviews.

### 5.4 Timing and interaction fluidity

The latency experienced between the initiation of a user request and the receipt of the corresponding system response is a critical factor in human–robot interaction, as delays have been shown to strongly influence how users perceive the naturalness and fluidity of LLM-driven systems. To address this, we measured the average processing time of each module in the pipeline, including the large language model (LLM), text-to-speech (TTS), automatic speech recognition (ASR), emotion recognition (EMO), and the gesticulation (GAN) modules. Table 6 presents a detailed breakdown of these waiting times across 10 interactions with Pepper.

**TABLE 6:** Average processing times (in seconds) per module for different stages of story narration during 10 interactions with Pepper.

	LLM	TTS	ASR	EMO	GAN	Total
Story selection	2.64	3.60				6.24
Setup	5.42	2.52	0.54		0.38	8.86
Confrontation		2.16	0.54		0.33	3.03
Resolution		3.24	0.54		0.49	4.27
Modified story narration	9.84	11.88	0.54	1.24	1.81	25.31

Notably, the LLM was invoked only once to generate the complete story, and subsequent segments corresponding to the Setup, Confrontation, and Resolution were buffered for offline deployment, preventing additional delays during storytelling. Nevertheless, the initial generation of the modified story required the longest latency ( ≈25 s), primarily due to the combination of LLM output length and TTS processing. In contrast, modules such as ASR and the GAN exhibited stable performance regardless of segment length or gesture complexity.

From a user experience perspective, shorter latencies ( ≈3 –8 s) during story selection, Setup, and Confrontation were generally tolerated, whereas longer delays, particularly during the modified story narration, were more noticeable. This aligns with previous findings that response time directly affects how engaging and “human-like” an interaction feels. Importantly, several participants noted in interviews that pauses were acceptable when framed as the robot “thinking,” but excessive waiting risks reducing immersion and disrupting the narrative flow.

## 6 Discussion

The performance of the robot as a collaborative storyteller can be appreciated in the video^4^. The video demonstration highlights both the strengths and limitations of the proposed system. It shows how the robot provides feedback to guide user interaction, such as audio tones and LED signals that indicate when it is listening. The robot’s expressiveness is also evident: while the original story is told in a neutral manner, the modified story includes gestures tied to specific words (e.g., branches (4:44, 4:50, 5:31), all (5:09), elephant (5:44, 5:59, 6:42), no (5:52) and thought (6:11) and emotions conveyed through speech modulation and LED color changes. These multimodal features enhance the storytelling experience by making it feel more dynamic and engaging.

However, some limitations were observed. The speech recognition module occasionally misidentified words (e.g., confusing “girl” with “guy”), which affected the accuracy of user inputs. Additionally, delays were noticeable during the generation of new stories, particularly at the modification stage, which may reduce the perceived fluidity of the interaction. While some participants tolerated pauses by interpreting them as the robot “thinking”, excessive latency risks breaking immersion.

Feedback from the affinity diagram analysis was largely positive. Participants valued the robot’s gesturing, emotional expression, and narrative adaptation, although a few noted abrupt gestures or sudden vocal modulations. These issues were seen as minor, suggesting that fine-tuning could further improve naturalness. Interaction design was also well received, especially the use of physical objects and multiple communication channels. While some participants desired clearer guidance, others found the system intuitive, pointing to a trade-off between simplicity and detailed instructions depending on user expertise.

It is important to stress, however, that this is a preliminary study, and the results should be interpreted with caution. The current evaluation does not include a comparison baseline, meaning we cannot yet conclude whether the proposed approach is objectively better than alternative methods. Narrative diversity was also limited: although the LLM produced 125 proposals across 25 interactions, only 23 unique titles appeared, with three stories dominating more than half of the suggestions. This bias toward familiar titles suggests the need for alternative prompting strategies or controlled story assignments to ensure greater variety. In addition, some participants noted that interacting in a non-native language restricted their spontaneity and creativity, underscoring the importance of supporting multiple languages in future versions.

Moreover, the relatively small sample size (N=25) in this study may have contributed to the instability of the Cronbach’s alpha estimates, as reliability coefficients are known to be highly sensitive to sample size. As recommended in the UEQ handbook, results from small samples (below 30–40 participants) should be interpreted with caution, and potential sampling effects considered when evaluating internal consistency. Therefore, the negative alpha observed for the perspicuity scale should not be overinterpreted, and these results are reported as exploratory.”

## 7 Conclusion and further work

This study presents a preliminary step toward multimodal collaborative storytelling with social robots. Pepper’s integration of natural language interaction, object recognition, gesture generation, and emotional expression demonstrated the feasibility of combining existing technologies into a unified storytelling framework. User feedback confirmed the novelty and appeal of the system, although several limitations remain regarding naturalness, latency, and narrative diversity. It is important to stress that this is a preliminary study, and the results should be interpreted with caution, since no comparison baseline was included.

In the context of storytelling, achieving high expressiveness across multiple modalities is crucial. While Pepper’s gesticulation and voice intonation generally aligned with the narrative, its expressive range is limited by the absence of facial expressions beyond LED modulation. More advanced gesture models, such as the Semantic Gesticulator (Zhang Z. et al., 2024), could generate semantically relevant movements, but their computational cost currently does not permit real-time execution. Similarly, although fine-tuned LLMs typically yield superior results, this process is resource-intensive. A zero-shot approach, as explored here, proved to be a viable alternative, and the continuous release of new LLMs opens the door to more creative, flexible, and open-ended interactions.

Future work should directly address the lack of a comparison baseline. Systematic evaluations are needed to compare Pepper narrating with and without gestures or tonal modulation, telling stories alone versus co-creating them with the user, and collaborative storytelling against a human narrator. Such studies would clarify the role of each expressive channel and assess the extent to which robots could complement, or eventually substitute, human facilitators, especially in educational or therapeutic contexts for children. Follow-up studies will also investigate why the LLM favored certain stories repeatedly, testing different prompting strategies to mitigate this bias or determine whether it reflects inherent model tendencies.

Finally, expanding evaluations to include larger and more age-diverse participant groups, ideally in their native language, will provide more representative feedback and reveal whether our work should be redirected to better address the needs and experiences of younger children.

## Data Availability

The original contributions presented in the study are included in the article/supplementary material, further inquiries can be directed to the corresponding author.

## Footnotes

1
https://huggingface.co/TheBloke/Llama-2-13B-AWQ

2
https://openai.com/index/whisper/

3
https://docs.ultralytics.com/models/yolo11/

4 Demo: https://youtu.be/u74-QV5LFD0
